# Severe mental illness and pregnancy outcomes in Australia. A population-based study of 595 792 singleton births 2009–2016

**DOI:** 10.1371/journal.pone.0264512

**Published:** 2022-02-28

**Authors:** Kristina Edvardsson, Elizabeth Hughes, Beverley Copnell, Ingrid Mogren, Don Vicendese, Richard Gray

**Affiliations:** 1 School of Nursing and Midwifery, La Trobe University, Bundoora, VIC, Australia; 2 Judith Lumley Centre, La Trobe University, Bundoora, VIC, Australia; 3 Faculty of Medicine and Health, School of Healthcare, University of Leeds, Leeds, United Kingdom; 4 Department of Clinical sciences, Obstetrics and Gynaecology, Umea University, Umea, Sweden; 5 The Department of Mathematics and Statistics, La Trobe University, Bundoora, Victoria, Australia; 6 The Melbourne School of Population and Global Health, University of Melbourne, Carlton, Victoria, Australia; Sabzevar University of Medical Sciences, ISLAMIC REPUBLIC OF IRAN

## Abstract

**Background:**

Women with Severe Mental Illness (SMI) may have more complex pregnancies and pregnancy outcomes that require different care and management, but this has not been extensively studied. The aim of this study was to explore associations between SMI and adverse maternal and infant outcomes in the state of Victoria, Australia.

**Methods:**

Our sample included all reported live singleton births in Victoria 2009–2016 (N = 595 792). Associations between SMI and adverse pregnancy outcomes were explored using Odds Ratios (OR), adjusted for sociodemographic and lifestyle factors, and co-morbidities, including any other mental illness.

**Results:**

Of all singleton births, 2046 (0.34%) were to a mother diagnosed with a SMI. We found evidence of an association between SMI and a range of adverse maternal and infant outcomes. Compared to women without SMI, women with a SMI had higher adjusted odds of being admitted to a High Dependency Unit or Intensive Care Unit (aOR 1.83, 1.37–2.43), having gestational diabetes mellitus (1.57, 1.34–1.84), undergoing an unplanned caesarean section (1.17, 1.02–1.33), induction of labour (1.17, 1.05–1.30) and postpartum haemorrhage (1.15, 1.03–1.29). Newborns of women with SMI had higher adjusted odds of being admitted to Special Care Nursery (aOR 1.61, 1.43–1.80), a low Apgar score at 5 minutes (1.50, 1.19–1.90), preterm birth (1.40, 1.20–1.63), and low birthweight (1.26, 1.06–1.49).

**Conclusion:**

Women with SMI are at higher risk for a range of adverse maternal and infant outcomes and are a population that may benefit from targeted early identification and enhanced antenatal care.

## Introduction

The health status of women prior to them becoming pregnant and their health behaviors during pregnancy are established predictors for pregnancy outcomes [1]. People with Severe Mental Illness (SMI)–schizophrenia, bipolar disorder and major depressive disorder–more commonly have a complex set of risk factors for ill health and excess comorbidity [2–4], which is mirrored in an estimated 8–17.5 year mortality gap between people with SMI and the general population [5–7].

Little attention has been given to the reproductive health of women with SMI. Historically, fertility rates have been low among women with SMI such as schizophrenia [8]. Some authors argue there has been little change in fertility rates over time for women with SMI [9]. However, there is emerging evidence suggesting an increase in fertility rates in women with schizophrenia, that are comparable with the general population [10]. The apparent increase in fertility has been attributed to factors including transition from institutional care to community-based care [11] and use of novel psychiatric medications with lower hormonal side effect profiles [12].

Pregnant women with SMI have increased rates of medical co-morbidities that can impact on pregnancy outcomes compared to women without SMI [13–16]. Obesity and smoking are among the most important modifiable risk factors for adverse maternal and child health outcomes [17], both overrepresented among women with SMI [13–15]. Women with SMI are also more likely to report an unintended pregnancy than those in the general population [8,18,19]. Further, discussions relating to contraception and pregnancy with women with SMI is not embedded in routine mental health care [20,21], and mental health staff report a lack of confidence in sexual and reproductive health counselling [22]. Consequently, women with SMI are likely to miss out on important advice on contraception, as well as pre-conceptual health interventions such as quitting smoking, reducing body mass index, regular physical exercise and a healthy diet, including vitamin and mineral supplements, safe food handling, and abstaining from alcohol and drugs [1].

While there is increasing research and clinical focus on the impact of common mental disorders–depression and anxiety–on pregnancy outcomes [23], research specific to pregnant women with SMI is still scarce. Since the 1960s [24], evidence has slowly accumulated about important associations between SMI and adverse pregnancy outcomes. However, many studies are small, few have considered known risk factors such as Body Mass Index (BMI), and prevalence estimates in pregnancy populations are rare. An increased understanding of patterns of associated health and lifestyle factors, comorbidities and pregnancy outcomes for women with SMI can guide preventative efforts, including a more targeted pre-conceptual care.

### Aim

The aim of this study was to explore associations between SMI (schizophrenia, bipolar disorder and severe depression), health and lifestyle factors and adverse maternal and infant health outcomes using a population-based sample of all reported singleton births in the state of Victoria, Australia 1999–2016. Additionally, we aimed to estimate the prevalence of documented SMI in this population.

## Methods

### Study design

A population-based cross-sectional study of 595 792 singleton births. Our reporting is compliant with STROBE guidelines for observational research.

### Setting and participants

Data were obtained from the Victorian Perinatal Data Collection (VPDC), a population-based surveillance system established in 1982 that collects data on maternal characteristics, intercurrent diseases and obstetric conditions, procedures and health outcomes for every birth in the state of Victoria, Australia [25]. Our sample included all live singleton births and stillbirths ≥ 20 weeks of gestation or with birth weight ≥400 g in cases where gestation was unknown, for the time period 2009–2016 (N = 595 792).

### Variables

#### Categorisation of variables based on ICD-10 codes

The VPDC database includes several variables where diagnoses are entered using International Statistical Classification of Diseases and Related Health Problems, Australian Modification (ICD-10-AM) codes [26]. This classification was used throughout the study period. We used the method described by Davey et al. [27] to identify diagnoses of interest across variables and fields within each variable (i.e. more than one ICD code can be recorded for each case), and recoded into a condition specific variable (e.g. SMI yes/no). The ICD-10-AM is an expanded version of the World Health Organization’s (WHO) ICD-10, and codes of interest in this study were either exactly the same, or could be mapped to the code immediately above it in the hierarchy of both classifications (e.g. the ICD-10-AM codes O24.42, O24.43, O24.44 and O24.49 were mapped to ICD-10 O24.4 ‘Diabetes mellitus arising in pregnancy’). None of the codes used in this study were mapped to different concepts between the classifications.

#### Outcomes

Maternal and infant health outcomes were coded as dichotomous categorical variables. *Maternal outcomes* were: admission to High Dependency Unit (HDU) or Intensive Care Unit (ICU); pre-eclampsia or eclampsia (ICD-10 O14-15); uterine inertia (inadequate contractions) (ICD-10 O62.0–62.2), postpartum haemorrhage (≥500 ml), gestational diabetes mellitus (ICD-10 O24.4, O24.9), induction of labour, planned caesarean section, and unplanned caesarean section.

*Infant outcomes* were low Apgar at 5 minutes (<7); stillbirth (the birth of an infant of at least 20 weeks’ gestation or if gestational age unknown, weighing at least 400 grams, which shows no signs of life after birth); neonatal death (within 28 days of birth); preterm birth (before 37 completed weeks of gestation); low birthweight (birth weight <2500 grams); small for gestational age (SGA), large for gestational age (LGA), birthweight ≥4000 grams, congenital anomaly (whether any congenital anomaly was identified), admission to Special Care Nursery (SCN); and admission to Neonatal Intensive Care Unit (NICU).

#### Exposures

The exposure variable SMI was coded as a dichotomous variable. Our definition of SMI included ICD-10 codes F20-F29 (e.g schizophrenia, persistent delusional disorder, schizoaffective disorder), F30-31 (bipolar disorder), and F32.3, F33.3 (depressive disorder with psychotic features). Our definition did not include patients with major depressive disorder (F32-F39) *without* psychotic features [28]. We note this is an area of contention and some authors have argued for a more inclusive definition of SMI (e.g. Ruggeri et al. [28]). The unexposed group was women without SMI.

#### Confounders

Based on our review of previous observational studies [13,29–33] and in consultation with expert clinicians, we generated a list of potential confounding variables that were included in the VPDC dataset. These included maternal characteristics, sociodemographic factors, and co-morbidities: age in years (continuous variable), marital status (dichotomised as ‘in a couple relationship’ [married or defacto] and ‘not in a couple relationship’ [single, divorced, widowed or separated]), country of birth (dichotomised Australia or rest of the world), Index of Relative Socioeconomic Disadvantage (IRSD) quintiles (based on postcodes, summarises variables that indicate relative disadvantage and ranks areas from most to least disadvantaged) [34], parity (total number of previous pregnancies that have resulted in a live birth or stillbirth, categorised into 0, 1, 2, and 3+), gestational age at 1^st^ antenatal visit (weeks), smoking status (maternal smoking behaviours before 20 weeks of pregnancy, coded as never smoked during pregnancy, stopped smoking, or continued smoking), Body Mass Index (BMI) categorised into underweight (<18.5), normal weight (18.5–24.9), pre-obesity (25.0–29.9), obesity class I (30–34.9), obesity class II (35–39.9) and obesity class III (≥40) according to the World Health Organization classification [35] (continuous BMI was used for descriptive purposes). Comorbidities included pre-existing diabetes mellitus (ICD-10 E10-E14 and O24.0-O24.3), gestational diabetes mellitus (ICD-10 O24.4, O24.9), hypothyroidism (ICD-10 E00-E03), hyperthyroidism (ICD-10 E05-E06), hypertensive disorder (excluding preeclampsia and eclampsia) (ICD-10 I10-I15, O10-O13, O16), asthma and other chronic obstructive pulmonary disease (ICD-10 J44-J46), and any other mental illness than SMI in which we included ICD-10 codes F00-F99 (excluding SMI diagnoses F20-F29, F30-31, F32.3, F33.3) and ICD-10 O993 (Mental disorders and diseases of the nervous system complicating pregnancy, childbirth and the puerperium).

#### Bias

The validity of the VPDC dataset has been established in two previous validation studies [27,36]. Authors of a validation study reported 100% specificity for key dichotomous variables used in this study: gestational diabetes mellitus, pre-eclampsia, pre-existing hypertension, pre-existing diabetes mellitus type 2 [36]. Accuracy (perfect match between medical record and VPDC data) was high (90%-100%%) for child’s date of birth, postcode, plurality, birth status, maternal age, maternal admission to HDU/ICU, parity, mode of delivery, Apgar at 5 minutes, birth weight, estimated gestational age, marital status, and mother’s country of birth. Smoking before 20 weeks (77%), maternal self-reported height (76%) and weight (64%) were reported to demonstrate sufficient accuracy.

#### Missing values

In this study, missing values were ≤0.1% for the following variables: maternal age, country of birth, postcode, parity, labour type, mode of delivery, HDU/ICU admission–mother, birthweight, gestational age; <2% for marital status, 1^st^ Antenatal visit (gestational age in weeks), smoking before 20 weeks of gestation, blood loss at delivery, Apgar at 5 minutes, birth status (complete variable), congenital anomaly indicator; 2.8% for SCN/NICU admission–baby (2.8% and 1.8% in the non-SMI and SMI groups, respectively); and 8.8% for BMI (8.8% and 14.0% in the non-SMI and SMI groups, respectively).

#### Implausible values

BMI values lower than 14 and above 60, and ages below 10 and over 60 years were considered implausible and excluded.

### Statistical analyses

Univariate analyses were undertaken which included comparisons of group means through independent samples t-tests, and exploration of differences between categorised variables through Chi-square tests (with Yates Continuity Correction for 2 by 2 tables). Logistic regression analyses were then undertaken to estimate crude Odds Ratios (OR) and their 95% Confidence Intervals (CI) for associations between SMI and maternal and infant outcomes, respectively. Multiple regression analysis was used to adjust for demographic and clinical characteristics. IBM SPSS Statistics 26 was used in all analyses.

### Ethical considerations

La Trobe University Ethics committee approved this study (reference S15/232). Use of the VPDC data was approved by the Consultative Council on Obstetric and Paediatric Mortality and Morbidity (CCOPMM) in Victoria, Australia.

## Results

### Demographic and clinical characteristics of participants

There were 595 792 singleton infants born in Victoria between 2009 and 2016, of which 2046 (0.34%) were to a mother with a SMI: 29% had an ICD F20-F29 [schizophrenia and related disorder] and 71% ICD F30-F31 [mood disorders] diagnosis.

Demographic and clinical characteristic of mothers giving birth in Victoria 2009–2016 are shown in Table 1. Women with a SMI were five times more likely to continue smoking whilst pregnant (42.5% vs 8.3%), more likely to be obese and more commonly diagnosed with diabetes mellitus (pre-existing and gestational), hypertensive disorder and asthma, the last condition with a 2.5 times higher prevalence in the SMI group (16.9% vs 6.8%). Women with SMI were also more likely to have a co-morbid psychiatric diagnosis (56.3% versus 8.8%).

**Table 1:** Demographic and clinical characteristics of women with and without severe mental illness giving birth in Victoria 2009–2016 (n = 595,792).

	Women with SMI		Women without SMI		P-value
N	2,046		593,746		
	n	%	n	%	
**Age, mean (SD)**	29.9 (6.2)		30.9 (5.4)		<0.001^1^
≤19	88	4.3	12 546	2.1	
20–24.9	368	18.0	63 377	10.7	
25–29.9	527	25.8	157 007	26.4	
30–34.9	531	26.0	211 132	35.6	
≥35	532	26.0	149 337	25.2	
**Marital status**					<0.001^2^
In a couple relationship	1,177	58.9	514 506	88.0	
Not in a couple relationship	820	41.1	70 299	12.0	
**Country of birth**					<0.001^2^
Australia	1,758	86.6	390 767	66.6	
Elsewhere	273	13.4	195 892	33.4	
**IRSD (Index of Relative Socioeconomic Disadvantage)**					<0.001^2^
Quintile 1 (most disadvantaged)	466	22.8	88 352	14.9	
Quintile 2	432	21.1	95 588	16.1	
Quintile 3	432	21.1	128 158	21.6	
Quintile 4	418	20.4	135 725	22.9	
Quintile 5 (least disadvantaged)	297	14.5	145 466	24.5	
**Parity**					<0.001^2^
0	870	42.6	262 466	44.2	
1	572	28.0	207 626	35.0	
2	315	15.4	80 888	13.6	
3+	287	14.0	42 650	7.2	
**1st antenatal visit, gestational age in weeks, mean (SD)**	15.97 (8.75)		13.95 (7.29)		<0.001^1^
**Smoking**					<0.001^2^
Never smoked	1,052	53.2	523 425	89.6	
Stopped smoking before 20 weeks’ gestational age	86	4.3	12 570	2.2	
Continued smoking before 20 weeks’ gestational age	840	42.5	48 482	8.3	
**BMI** ^**3**^	27.89 (6.64)		25.85 (5.72)		<0.001^2^
<18.5 Underweight	54	3.1	16 657	3.1	
18.5–24.9 Normal weight	632	35.9	274 822	50.8	
25.0–29.9 Pre-obesity	516	29.3	144 364	26.7	
30.0–34.9 Obesity class I	312	17.7	64 198	11.9	
35.0–39.9 Obesity class II	142	8.1	26 254	4.8	
≥40 Obesity class III	104	5.9	15 192	2.8	
**Comorbidities**					
Pre-existing diabetes mellitus	28	1.4	4 688	0.8	0.005^2^
Gestational diabetes mellitus	224	10.9	47 704	8.0	<0.001^2^
Hypothyroidism	42	2.1	14 409	2.4	0.305^2^
Hyperthyroidism	15	0.7	3 491	0.6	0.476^2^
Hypertensive disorder (excl preeclampsia and eclampsia)	109	5.3	22 184	3.7	<0.001^2^
Asthma and other chronic obstructive pulmonary disease	345	16.9	40 336	6.8	<0.001^2^
Any mental illness (except SMI)	1,151	56.3	52 484	8.8	<0.001^2^

^1^Independent samples t-test

^2^Chi-square tests (with Yates Continuity Correction for 2 by 2 tables)

^3^WHO classification (35).

Mothers with SMI were on average one year younger (mean 29.9, range 14–46) than women without SMI (mean 30.9, range 10–59), were more likely to be single, divorced, widowed or separated (41.1% vs. 12.0%), born in Australia (86.6% vs. 66.6%), and living in areas with greater social disadvantage. Women with a SMI visited antenatal care for the first time on average two weeks later than women without SMI.

### Association of SMI with maternal and neonatal outcomes

#### Maternal outcomes

Having an SMI diagnosis increased the odds of a range maternal outcomes (Table 2) even after adjustment for maternal age, parity, smoking, SMI, marital status, country of birth, Index of Relative Socioeconomic Disadvantage (IRSD), and a range of co-morbidities, including any other mental illness. In our adjusted model, women with SMI had 83% higher odds of being admitted to a higher dependency unit (HDU) or Intensive care Unit (ICU), 57% higher odds of gestational diabetes, 17% higher odds of being induced and having a caesarean section, respectively, and 15% higher odds of postpartum haemorrhage, compared with women without SMI. The strength of associations decreased between the crude and adjusted model across all variables, except gestational diabetes, which instead increased in strength (cOR 1.41, CI 1.23–1.62; aOR 1.57, CI 1.34–1.84). A 42% increase in the odds of preeclampsia/eclampsia was found in the crude model; however, this association disappeared after adjustment. No associations between SMI and dystocia and planned caesarean sections were found (Table 2).

**Table 2:** Association between severe mental illness and maternal outcomes in Victoria 2009–2016.

Outcome	Prevalence, % (n)				Odds Ratio (OR) for adverse maternal outcomes for women with SMI					
			Crude OR 2009–2016				Adjusted OR 2009–2016^1^			
	SMI	No SMI	Included in analysis (n)	Crude OR	95% CI	P-value	Included in analysis (n)	OR	95% CI	P-value
Admission to High Dependency Unit (HDU) or Intensive care Unit (ICU)	3.1 (63)	1.4 (8 169)	594 948	2.28	1.77–2.93	<0.001	521 255	1.83	1.37–2.43	<0.001
Preeclampsia/eclampsia^2^	3.4 (70)	2.4 (14 475)	595 792	1.42	1.12–1.80	0.004	521 880	1.02	0.77–1.35	0.884
Uterine inertia (ICD-10 O62.0–62.2)	5.8 (119)	6.1 (36 237)	595 792	0.95	0.79–1.14	0.588	521 880	1.09	0.89–1.33	0.424
Postpartum haemorrhage (≥500 ml)	25.2 (509)	22.3 (130 370)	587 935	1.18	1.06–1.30	0.002	515 956	1.15	1.030–1.29	0.014
Gestational diabetes mellitus^3^	10.9 (224)	8.0 (47 704)	595 792	1.41	1.23–1.62	<0.001	521 880	1.57	1.34–1.84	<0.001
Induction of labour	33.2 (680)	26.9 (159 873)	595 651	1.35	1.23–1.48	<0.001	521 789	1.17	1.05–1.30	0.005
Planned caesarean section	16.6 (340)	16.7 (99 254)	595 592	0.99	0.88–1.12	0.899	521 749	1.06	0.92–1.21	0.442
Unplanned caesarean section	18.5 (379)	15.4 (91 275)	595 592	1.25	1.12–1.40	<0.001	521 749	1.17	1.02–1.33	0.021

Logistic regression.

^1^Adjustment factors: Maternal age, Parity, Smoking, BMI, Marital status, Country of birth, IRSD (Index of Relative Socioeconomic Disadvantage), Pre-existing diabetes mellitus, Gestational diabetes mellitus, Hypothyroidism, Hyperthyroidism, Hypertensive disease, Preeclampsia and eclampsia, Asthma and other chronic obstructive pulmonary disease, Any other mental illness except SMI.

^2^Preeclampsia/eclampsia excluded in Adjusted OR.

^3^Gestational diabetes mellitus excluded in Adjusted OR.

#### Infant outcomes

After adjusting for confounding, we observed associations between women with SMI and a 61% higher odds of the infant being admitted to Special Care Nursery (SCN), a 50% higher odds of low Apgar at 5 minutes, a 40% higher odds of preterm birth, a 26% higher odds of low birthweight and a 21% higher odds of the infant being born with congenital anomalies (borderline association). In the unadjusted model, associations were found also between SMI and being Small for Gestational Age (SGA),being admitted to a Neonatal Intensive Care Unit (NICU), and a lower odds of a birthweight ≥4000 g. There were no associations between SMI and stillbirth, neonatal death, and being Large for Gestational Age (LGA), respectively, in either the unadjusted or adjusted models (Table 3).

**Table 3:** Association between severe mental illness and infant outcomes in Victoria 2009–2016.

Outcome	Prevalence, % (n)				Odds Ratio (OR) for adverse neonatal outcomes for women with SMI					
			Crude OR 2009–2016				Adjusted OR 2009–2016^1^			
	SMI	No SMI	Included in analysis (n)	Crude OR	95% CI	P-value	Included in analysis (n)	OR	95% CI	P-value
Low Apgar Score (<7)	4.9 (100)	2.1 (12 673)	594 424	2.36	1.93–2.89	<0.001	520 797	1.50	1.19–1.90	0.001
Stillbirth	0.6 (12)	0.4 (2 671)	595 792	1.31	0.740–2.31	0.358	521 880	1.22	0.67–2.23	0.510
Neonatal death	0.1 (3)	0.2 (1 142)	595 792	0.76	0.25–2.37	0.638	521 880	0.83	0.26–2.60	0.748
Preterm birth	13.1 (268)	6.4 (37 915)	595 189	2.21	1.94–2.51	<0.001	521 383	1.40	1.20–1.63	<0.001
Low birthweight	10.6 (217)	5.1 (30 159)	595 008	2.22	1.93–2.55	<0.001	521 304	1.26	1.06–1.49	0.009
Small for Gestational Age (SGA)	13.2 (271)	9.0 (53 426)	595 598	1.54	1.36–1.76	<0.001	521 720	1.06	0.91–1.24	0.436
Large for Gestational Age (LGA)	10.0 (204)	10.9 (64 833)	595 792	0.90	0.78–1.04	0.170	521 880	0.93	0.79–1.10	0.391
Birthweight ≥4000 g	9.2 (188)	11.7 (69 231)	595 008	0.77	0.66–0.89	0.001	521 304	0.90	0.76–1.06	0.199
Congenital anomaly	6.2 (127)	4.3 (24 986)	589 753	1.50	1.25–1.79	<0.001	516 237	1.21	0.98–1.50	0.081
Admission to Special Care Nursery (SCN)	28.1 (565)	13.3 (77 040)	579 402	2.54	2.31–2.80	<0.001	506 441	1.61	1.43–1.80	<0.001
Admission to Neonatal Intensive Care Unit (NICU)	3.4 (69)	1.5 (8 374)	579 402	2.42	1.90–3.08	<0.001	506 441	1.26	0.89–1.78	0.194

Logistic regression.

^1^Adjustment factors: Maternal age, Parity, Smoking, BMI, Marital status, Country of birth, IRSD (Index of Relative Socioeconomic Disadvantage), Pre-existing diabetes mellitus, Gestational diabetes mellitus, Hypothyroidism, Hyperthyroidism, Hypertensive disease, Preeclampsia and eclampsia, Asthma and other chronic obstructive pulmonary disease, Any other mental illness except SMI.

## Discussion

The aim of this study was to explore associations between Severe Mental Illness (SMI) and adverse maternal and neonatal outcomes in Victoria, Australia. Our findings show that having a diagnosis of SMI increased the odds of a range of adverse maternal and neonatal outcomes compared with women without SMI, and these remained significant after adjusting for confounders.

Our sample included all reported singleton births in Victoria during the time period, which eliminates the risk of response and sampling bias, and strengthens the external validity of the study [37]. We included a comprehensive set of confounders in our analyses, and to our knowledge this is the first study that also adjusts for the effect of additional mental illness in women with SMI. The VPDC dataset has shown high levels of accuracy through validation studies [25,27], and is considered appropriate for conservative analysis of relationships between variables [27]. Less than perfect accuracy for items has been explained by underreporting, rather than incorrect reporting to the VPDC of conditions that were not mentioned in the medical record [27]. This study has limitations. The level of underreporting of SMI in the study context is unknown, and it is possible that women with a SMI documented in the maternal record represented more complex cases. We did not have information about substance abuse or psychiatric medications used during pregnancy. These factors may have contributed to any residual confounding effects [32,38]. Furthermore, the number of comparisons made inherently carries a risk of false positive results. The cost of false positive (type I errors) was weighed against false negative (type II errors), and a decision was made not to adjust p-values since this study investigated distinct outcomes with focussed associations that were defined a priori [39].

A comparison of prevalence of SMI in pregnancy across populations is difficult due to a scarcity of such population based studies [12], as well as differences in data collection methods and definitions of SMI. Our study indicated a higher SMI prevalence when compared to a population-based study from Taiwan, where 0.064% of women with singleton births were identified with bipolar disorder, and 0.09% with schizophrenia [40]. Our prevalence was lower when compared to a recent study from the United States which identified a SMI diagnosis through maternal records in 0.79% of births. The definition of SMI in this US study included, in addition to bipolar disorder and schizophrenia, major depressive disorders which may partly explain this difference [13]. The ‘any mental illness’ prevalence in our study is largely comparable to findings from California, where a ‘mental illness diagnostic code’ prevalence of 8.5% was found in hospital discharge records for women who had a singleton live birth [41].

Our findings are also consistent with other studies showing a higher prevalence of risk factors and comorbidities among women with SMI, and poorer maternal and child health outcomes. Previous studies have identified higher prevalence of smoking [13–15,31–33,42,43], living alone or not being in a relationship [31,33], diabetes mellitus [13–16,43], hypertensive disorder [15,16], obesity [13,14] and asthma [14]; adverse maternal outcomes including caesarean section [13,15,16], induction of labour [15,16,44], intensive care unit admission [16], postpartum haemorrhage [15], preeclampsia [13,16]; and adverse neonatal outcomes including a low Apgar score at 5 minutes [32,42,43,45], infant low birthweight [30,31,40,45], and preterm birth [13–16,30–32,40,45], which supports our findings. Our observations are also consistent with previous findings from Australia of women with SMI presenting later for their first antenatal visit compared to women without SMI [42].

In contrast to the results of a large study from the United States based on a sample of 23 507 597 hospital births, we did not find an association between SMI and stillbirth [15]. The association between SMI and congenital anomalies weakened in the adjusted model in our study. This can possibly be explained by the inclusion of BMI among confounders, since congenital malformations have been shown to increase progressively with increasing overweight and obesity [46]. In the study by Zhong et al. in the United States, infants of women with SMI (schizophrenia, affective psychosis and other psychoses) had 49% higher adjusted odds of fetal abnormalities, and in a Danish study where 2 230 children of women with schizophrenia were compared with 123 544 children in the general population, children had a 70% increase in the risk of congenital malformations [47]. However, BMI was not included among the range of adjustment variables in these studies which may explain this discrepancy with our findings [15].

Previous studies from Australia have found higher rates of pre-eclampsia and gestational diabetes mellitus among women with SMI. These studies included small samples (112 and 138 respectively) [42,43]. In contrast to these findings, in our study the association between SMI and pre-eclampsia disappeared in the adjusted model, possibly due to the comprehensive set of adjustment factors.

The findings of this analysis support the development and testing of preconceptual counselling interventions that could be incorporated into in routine mental health care [12,48,49]. Ideally, interventions to address risk factors (such as smoking and obesity) need to occur prior to pregnancy, since preconceptual health as well as health status during pregnancy can predict maternal and child health outcomes. The contribution of potentially modifiable risk factors to adverse pregnancy outcomes have also been outlined in a study of women with schizophrenia in Ontario, Canada [32].

In our study, smoking was five times as prevalent and asthma more than twice as prevalent in women with SMI. These differences mirror findings from a study of 420 pregnant women with SMI in Western Australia [14]. Smoking is a highly prevalent [13–15,31–33,42] yet modifiable risk factor for women with SMI. Interestingly, a study from the UK found that pregnant women with a mental disorder were more likely than other pregnant smokers to accept referral to smoking cessation services; however, they were still more likely to smoke at delivery. Women received little support beyond the initial referral, and some women expressed that their care givers gave priority to mental health over smoking cessation [50]. Understanding barriers and facilitators for addressing modifiable risks for adverse pregnancy outcomes for women diagnosed with SMI seems imperative in efforts to improve health outcomes for mothers and their children.

### Conclusions and implications for practice

Maternal and infant health outcomes are significantly worse for women with SMI than for women without SMI. We identified high levels of smoking and women with SMI were more likely to be single parents, living in a more disadvantaged area, have higher BMI and prevalence of comorbidities and delayed contact with antenatal care. Low Apgar, preterm birth, and low birthweight indicate poor neonatal health, and this can have lifelong consequences for the offspring. Therefore, there is a need to address the health and wellbeing of women with SMI prior to pregnancy to optimize health outcomes for this overlooked and largely ignored population. It is noteworthy that the Lancet Commission on protecting physical health in people with mental illness does not substantially address sexual and reproductive health [51]. Our observations suggest that this is an important omission by the Commission.

**Key message**

The link between Severe Mental Illness (SMI) and adverse pregnancy outcomes has not been extensively studied using population-based data.

- Women with SMI were more likely to smoke, be single, live in a socially disadvantaged area, demonstrate higher BMI, have physical health comorbidities, and delayed contact with antenatal care.
- Having an SMI diagnosis increased the odds of a range of adverse maternal and infant outcomes, even after adjustment for key confounders.
- Enhanced preconceptual care for women with SMI may be effective in improving health outcomes for pregnant women and their babies.
